# Description of ROM-SPORT I Battery: Keys to Assess Lower Limb Flexibility

**DOI:** 10.3390/ijerph191710747

**Published:** 2022-08-29

**Authors:** Antonio Cejudo

**Affiliations:** 1 Department of Physical Activity and Sport, Faculty of Sport Sciences, CEIR Campus Mare Nostrum (CMN), University of Murcia, 30720 Murcia, Spain; antonio.cejudo@um.es; Tel.: +34-868-888-430; 2 Locomotor System and Sport Research Group (E0B5-07), University of Murcia, 30720 Murcia, Spain

**Keywords:** fitness, muscle flexibility, restricted range of motion, injury prevention, athletic performance

## Abstract

Limited range of motion (ROM) is considered one of the most important intrinsic and modifiable risk factors for the most common sports-related injuries. In addition, controlling and monitoring an athlete’s ROM is a strategy to achieve optimal ROM and improve athletic performance in sports, especially those that require high ROM in the major joints. Therefore, assessing ROM (pre-participation, during a rehabilitation process, on return to play, etc.) is important not only as a method to prevent sports injuries, but also as a quantitative determinant of the potential of athletic performance. However, despite the variety of different ROM assessment methods described in the literature, there is no consensus on which methods are best suited for this goal. Recently, the ROM-SPORT I battery has been shown to have advantages over other ROM assessment methods. This tool has not yet been fully described in detail for researchers, sports professionals, and clinicians to learn. The main objective of this study is to describe the ROM-SPORT I battery tests in detail using the following criteria: test description, simplicity of the test procedure, low need for human and material resources, predictive validity, and reliability.

## 1. Introduction

Sporting activities play an important role in our society today [1]. Millions of people around the world play some kind of sport (either at amateur or professional level) for many reasons, including enjoyment and fun, relaxation, socialisation, and maintaining or improving fitness and health. However, playing a sport also carries a certain risk of injury [2]. Previous epidemiological studies have shown that sports-related injuries account for 10–19% of all cases of acute injury in the emergency department [1,3]. 

Several research studies and centres of sports medicine and science emphasise the importance of regular pre-participation and in-season assessments to identify the primary and modifiable factors that may predispose athletes to injury. This is the most effective way to prevent and reduce the number and severity of sports injuries [2,4,5,6,7,8]. Identifying athletes at high risk of injury therefore allows for the implementation of specific interventions that directly target the critical factors related to the mechanisms of injury [6,8].

Limited range of motion (ROM) due to a lack of muscle flexibility has been shown to be one of the most important predictors of common sports injuries, such as groin pain (restricted hip abduction [9,10,11], lateral rotation [9], medial rotation [12,13], and total rotation [9] ROMs); tendinopathies of the patella (restricted hip flexion with the knee extended [10,12,14] and ankle dorsiflexion ROMs [15,16]) and Achilles (restricted ankle dorsiflexion ROM [17]); strains of the hamstrings (restricted hip flexion with the knee extended [18,19], knee flexion [18], and ankle dorsiflexion ROMs [20]) and quadriceps (restricted knee flexion ROM [18,19]); rupture of the anterior cruciate ligament (restricted lateral [21,22] and medial hip rotation ROMs [21,23,24]); low back pain (restricted hip flexion with the knee extended [25,26], hip extension [27], hip lateral rotation [28,29], hip internal rotation [26,29], and knee flexion [30] ROMs) and knee pain (restricted hip extension [31] and flexion ROMs [32]). In addition, certain sports such as taekwondo, diving, figure skating, and gymnastics require high ROM values in order for athletes to successfully perform the technical actions most highly rated by the judges [33] and improve their physical performance [34,35]. It therefore seems clear that ROM should be assessed in athletes as an intervention strategy to optimise their athletic performance and reduce their risk of injury. Flexibility has been shown to be specific to each joint, muscle action, or specific movement [25,26]. For this reason, the assessment of flexibility in athletes has shown very different results depending on the sport [36,37,38]. For example, artistic gymnastics, rhythmic gymnastics, and swimming jumps had the highest values for hip flexion with the knee extended ROM and the lowest values for water polo and marathons [38]. Shoulder and ankle ROM have higher values in speed swimmers than in other athletes [39]. Ultramarathon runners who cover a longer distance in competition maintain baseline values for hip flexion with the knee extended ROM and stride length during competition [40]. Goalkeepers have higher ROM values than outfield players only for knee flexion, hip flexion with the knee extended, and hip abduction [41,42]. The dominant limb of soccer players has higher values for knee flexion ROM than the non-dominant limb [43,44]. Rowers with more years of experience show higher values for hip flexion with the knee extended ROM than rowers with less experience and a lower competition level [45]. International dancers and gymnasts have higher ROM values in the shoulder (flexion and extension), hip (flexion, extension, and abduction), ankle (from dorsiflexion to planar flexion), and trunk (from hyperextension to full flexion) than national athletes and beginners [46]. International kickboxers have a higher ROM of hip adduction and ankle dorsiflexion than national fighters [47]. Medal athletes in Taekwondo show higher flexibility than non-medal athletes in passive and active hip flexion with the knee extended test and in the hip abduction test [48]. Furthermore, it has recently been shown that flexibility depends on age and maturation [49,50].

There is a large number of published tests to assess the ROM of the major joints of the lower extremities (hip, knee, and ankle). There are different methods of assessing ROM, e.g., passive (e.g., Straight Leg Raise Test (hip flexion ROM)) or active (e.g., Walking Step Test (ankle dorsiflexion ROM)), and/or using single (Thomas Test (hip extension ROM)) or multiple (Deep Back Squat (hip flexion ROM)) tests. In addition, numerous measuring instruments have been proposed to measure ROM directly (goniometer, inclinometer, etc.) or indirectly (tape measure, video camera, etc.) in degrees.

However, despite the large number of published ROM tests, there is currently no consensus on which examination tests are most appropriate for assessing the major joints of the lower limbs. Identifying criterion-referenced assessment tests and promoting their use in sports medicine and competitive sports would allow clinicians, physiotherapists, and sports professionals to standardise the assessment and monitoring of ROM. This would also facilitate the identification of athletes who are at risk of injury and/or whose ROM values are insufficient to achieve a higher level of technical athletic performance.

Knowledge of ROM monitoring can also lead to the application of training interventions to improve the athlete’s ROM values (e.g., stretching or foam rolling) [51]. In addition, it would be possible in research to more reliably investigate the role of ROM in the development of acute and overuse-related musculoskeletal pathologies associated with restricted ROM values (establishing normal and restricted cut-off values) and to support the efficacy of different treatments (e.g., stretch training, massage, or self-myofascial release exercises) to maintain and/or improve ROM [52,53]. For all these purposes, Hopkins et al. [54,55,56] suggests that the selection of diagnostic reference tests should first be based on the criteria of high validity and reliability, and then emphasise the simplicity and universality of the procedure.

Recently, the measurement method of the ROM-SPORT I battery tests has been shown to have advantages over other methods published in the literature [57]. However, no detailed description of the ROM-SPORT I battery tests has been published. Therefore, the main objective of this study is to describe the ROM-SPORT I battery tests in detail using the following criteria: test description, simplicity of the test procedure, low need for human and material resources, predictive validity, and reliability.

## 2. Description of the Tests of ROM-SPORT Battery I

**Figure 1:**
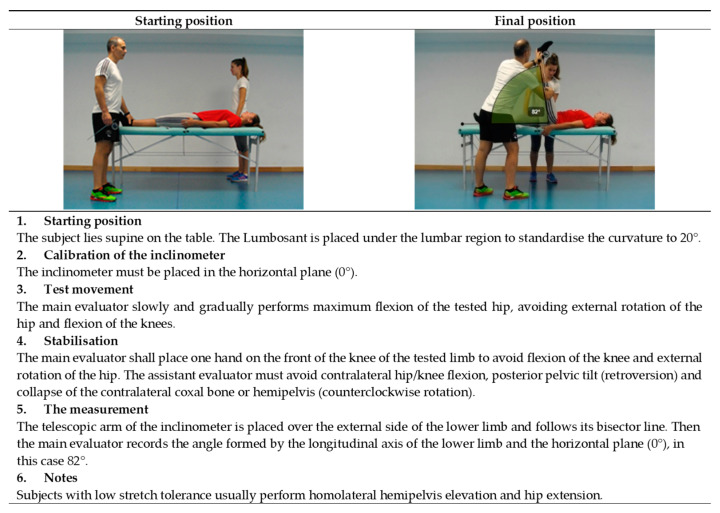
Passive hip flexion with the knee extended test (hamstrings).

**Figure 2:**
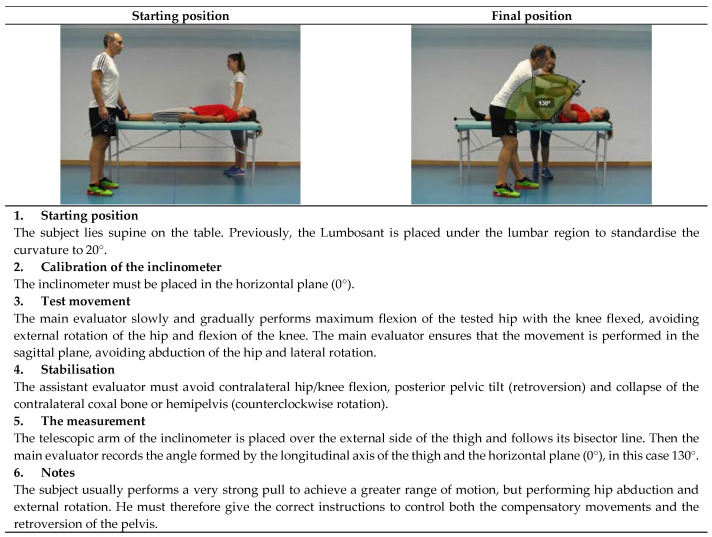
Passive hip flexion with the knee flexed test (gluteus maximus).

**Figure 3:**
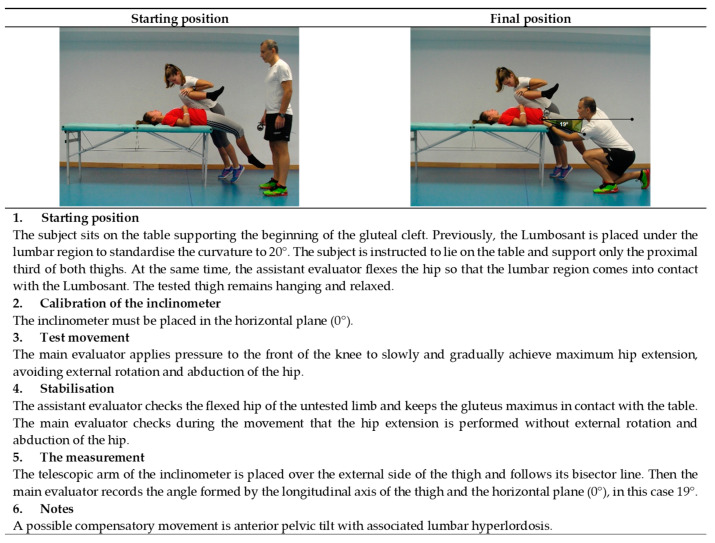
Passive hip extension with the knee relaxed test (iliopsoas).

**Figure 4:**
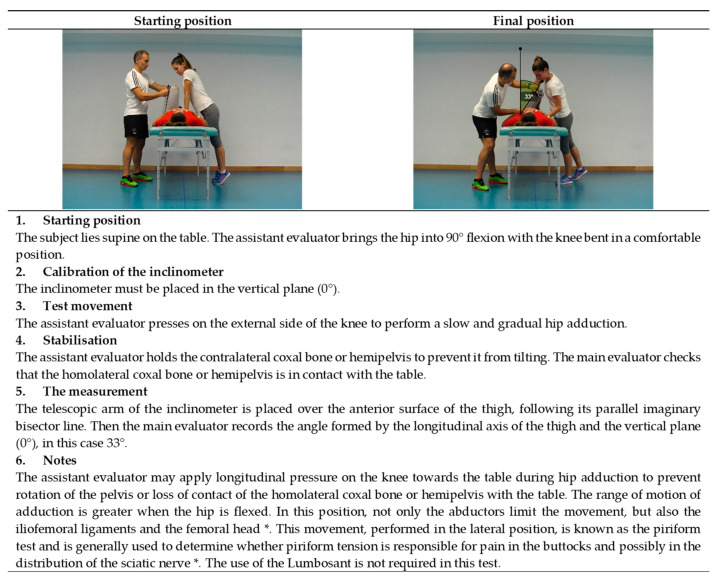
Passive hip adduction with the 90° hip flexion test (piriformis); * [59,60].

**Figure 5:**
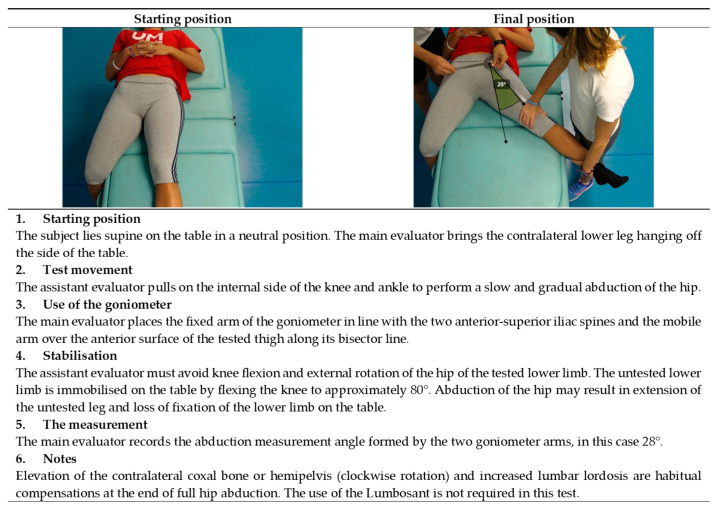
Passive hip abduction with the knee extended test (adductors).

**Figure 6:**
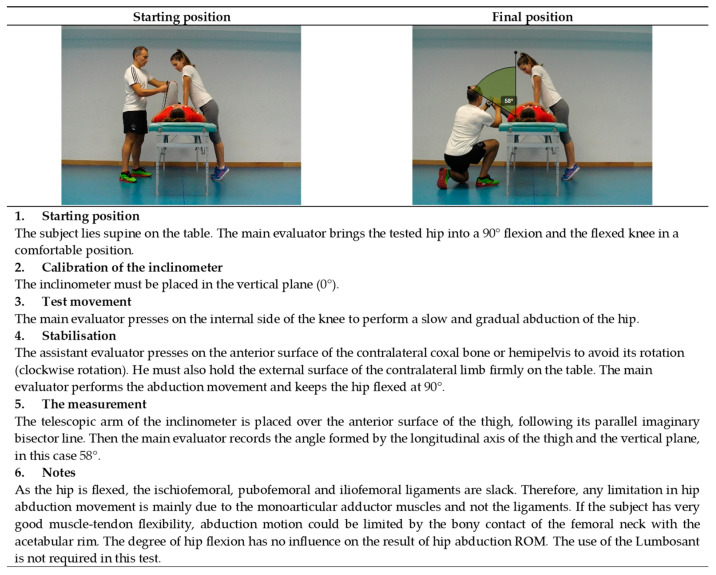
Passive hip abduction with the 90° hip flexion test (adductors monoarticular).

**Figure 7:**
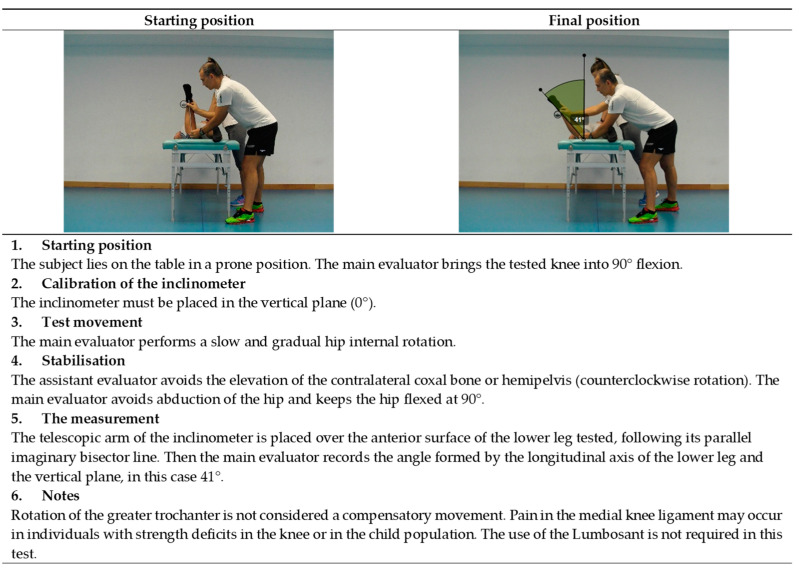
Passive internal hip rotation test (external rotators).

**Figure 8:**
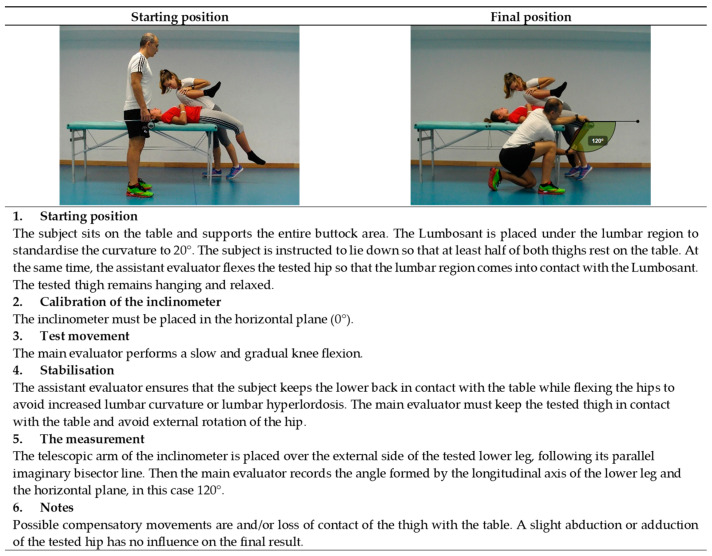
Passive external hip rotation test (internal rotators).

**Figure 9:**
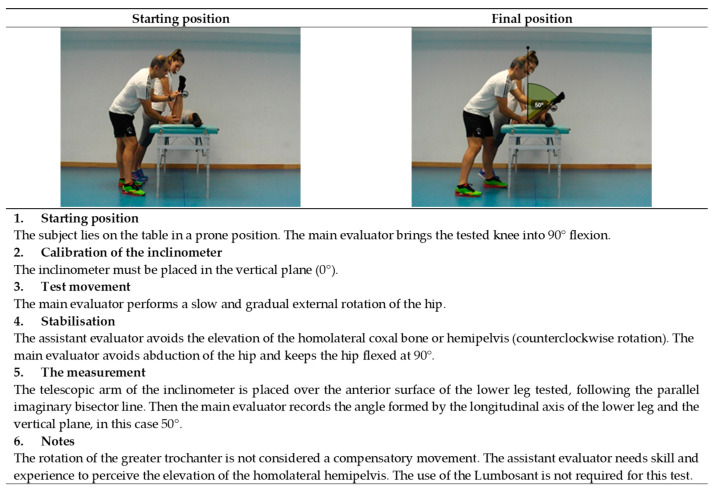
Passive knee flexion test (quadriceps).

**Figure 10:**
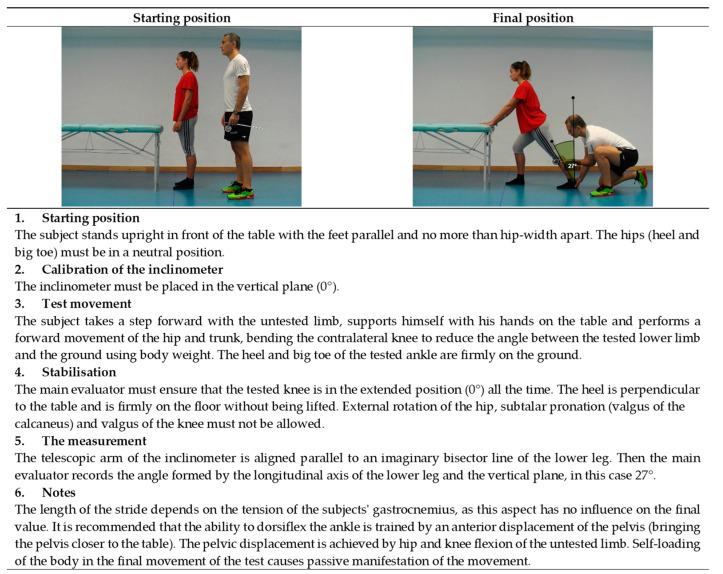
Ankle dorsiflexion with the knee extended test (gastrocnemius).

**Figure 11:**
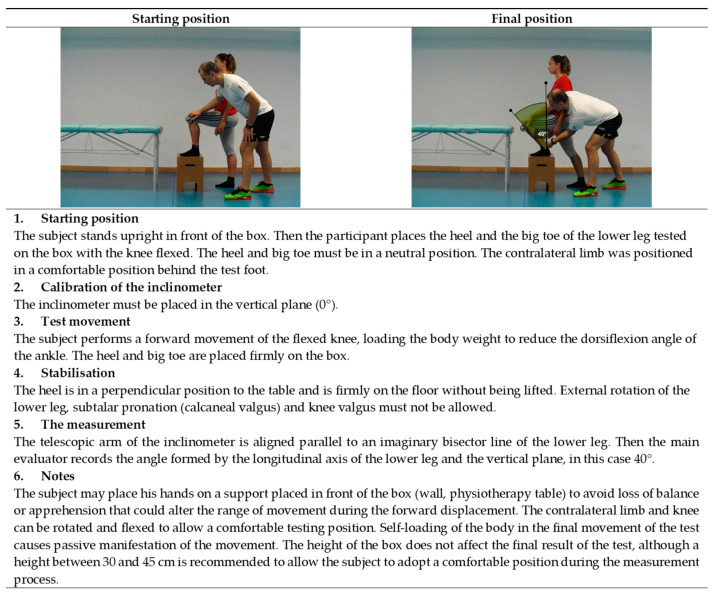
Ankle dorsiflexion with the knee flexed test (soleus).

The current study proposes to perform 11 assessment tests in the extended version of ROM-SPORT I battery to obtain the ROM measurements of the major joints of the lower extremities. The following ROM measurements would be taken at the hip: flexion with the knee extended (Figure 1), flexion with the knee flexed (Figure 2), extension with the knee relaxed (Figure 3), adduction with 90° hip flexion (Figure 4), abduction with the knee extended (Figure 5), abduction with 90° hip flexion (Figure 6), internal rotation with the knee flexed (Figure 7), and external rotation (Figure 8) with the knee flexed. In the knee, flexion is assessed (Figure 9), while in the ankle, dorsiflexion is measured with the knee extended (Figure 10) and flexed (Figure 11).

In the shortened version of the ROM-SPORT I battery, 7 tests are performed to obtain the following ROM measurements: in the hip, flexion with the knee extended and extension with the knee relaxed; in the knee, flexion; and in the ankle, dorsiflexion with the knee extended and flexed. These tests were chosen for the shortened version of ROM-SPORT I battery because they measure the ROMs most commonly reported as restricted in athletes [57] and the general population [36,58].

The procedure for each test in the ROM-SPORT I battery is described in detail in the following Figure 1, Figure 2, Figure 3, Figure 4, Figure 5, Figure 6, Figure 7, Figure 8, Figure 9, Figure 10 and Figure 11.

## 3. Highlights of the ROM-SPORT I Procedure

### 3.1. Familiarisation and Warm-Up Phase

Before assessing ROM, the athlete should perform 8–10 min of warm-up training with cardio (jogging or cycling; 50–70 W and 60–80 rpm; light intensity, 10–12 Borg scale) according to the recommendations [61,62] and 60–90 s of dynamic flexibility of the major lower limb muscles (gluteus maximus, hamstring, adductors, iliopsoas, quadriceps, external and internal rotators of the hip, gastrocnemius and soleus) [63]. Previous studies have reported that 60–180 s of static stretching produces a lasting change in the viscoelastic properties of muscles during a 20–30 min rest period [63]. The application of the ROM-SPORT I battery to an athlete takes less than 20 min [57]. The positions used for the stretching exercises should be similar to those used for the different tests to reduce possible learning bias in the results.

Warm-up exercises were performed because: (1) all tests required a large muscle tension stimulus; (2) warm-ups reduce the effects of muscle stretch by repeated trials during data collection; and (3) they reduce the variability and standard error of measurements by minimising the effects of different muscle temperatures on muscle flexibility [64,65].

After the warm-up, the ROM assessment is performed.

### 3.2. Human Resources

One of the disadvantages of the ROM-SPORT I battery is that the ROM assessment of the hip and knee joints requires two trained evaluators: one to ensure that the athlete remains in the correct position during the test manoeuvres (assistant evaluator) and another to perform the test (main evaluator). However, only one evaluator is required for the assessment of the ankle ROM. The use of two evaluators to perform the tests seems to limit the practical application of these measurement methods in a sporting and clinical context. As these measurement methods are simple to use, the role of the assistant evaluator (ensuring adequate stabilisation of the pelvis and other body segments during all tests) could be taken on by any postgraduate student or sports coach conducting one or two 10-min training sessions (statement based on the authors’ extensive experience).

### 3.3. Athlete’s Starting Position

The athlete’s starting position is the neutral or zero-degree position. Seven of the eleven tests of the ROM-SPORT I battery are performed in the supine position (hip and knee ROM), with the exception of the hip rotation and the ankle ROM tests. Therefore, depending on the position of the athlete, the tests can be performed in the following order: standing (ankle dorsiflexion ROM tests), prone (hip rotation tests), and supine (hip ROM tests), or the other way round. This order of ROM test performance helps to shorten the duration of the evaluation session. The competencies of the main and the assistant evaluator are brief and clearly defined. The main evaluator checks the correct starting position of the athlete and performs the target movement of the assessment, while the assistant examiner checks the compensatory movements with his hands and the Lumbosant.

Finally, a table and a standard box (about 30.5 cm high) are used to position the athletes for the tests. The use of a box to position the athletes for the ankle dorsiflexion with the knee flexed ROM test allows the evaluator and the athletes to adopt a comfortable position during the measurement procedure of the ankle dorsiflexion ROM [66].

### 3.4. Measuring Instrument and Its Calibration

The starting position of the athlete; the competences, capabilities, and skills of the evaluators; and the use of the measuring instrument lead to a simple and fast ROM measurement. An inclinometer (ISOMED, Inc, Portland, OR, USA) with a telescopic arm is used as the main measuring instrument for all ROM assessment tests, except for the hip abduction with the knee extended ROM test, which requires a long-arm goniometer. A lower-back protection support, or Lumbosant (Imucot Traumatología SL, Murcia, Spain), is used in the hip and knee ROM tests to standardise the lordotic curve (20°) during the assessment manoeuvres [67,68,69].

The use of an inclinometer with a telescopic arm has the advantage that no body landmarks need to be marked, since the maximum ROM values can be determined as the angle that the longitudinal axis of the moving body segment (imaginary line of the lateral bisector (sagittal movements) or anterior bisector (frontal movements) of the mobilised body segment) makes with the vertical or horizontal plane. Thus, the initial and final positions can be systematically and repeatedly determined with precision [58]. The inclinometer with a telescopic arm becomes a single-arm goniometer, which has the advantage of having a plane of gravity; this allows for better measuring accuracy and a higher measuring speed [57,69].

Depending on the movement, the inclinometer is calibrated with gravity at 0° vertically (adduction and abduction with 90° hip flexion; internal and external rotation with flexed knee; ankle dorsiflexion with extended and flexed knee) or horizontally (flexion with extended and flexed knee; extension with relaxed knee and knee flexion).

### 3.5. Movement of the Assessment

In the ROM-SPORT I battery procedure, a maximum of passive movement is used in all tests. The rationale for using passive manoeuvres is for the following two reasons: Firstly, in several active tests, the peak ROM depends on the athlete’s muscle strength (mainly psoas, hamstrings, quadriceps) and the ability to simultaneously contract the agonist muscles and relax the antagonist muscles being measured [70]. This severely limits the use of active testing in individuals with low physical conditions, such as children and adolescents [71]. Furthermore, the different strength levels of athletes of different sexes and different sports do not allow for a comparison of the flexibility profiles [72]. Secondly, the athlete’s motivation has been shown to influence the result of an active ROM test, leading to intraindividual variability or a source of measurement error [58].

For the ankle dorsiflexion tests ROM, the body weight itself should be loaded in order to obtain a maximum passive measurement [51]. In addition, all selected passive tests specifically measure the main movement of the joint, i.e., the extensibility of the target muscle. Linear and functional tests (e.g., shoulder mobility test, overhead squat test, floor-to-toe distance test) have more disadvantages than angular tests for assessing muscle extensibility. These tests are significantly influenced by the anthropometric measures and intermuscular coordination of the subjects, which negatively affects the validity of the results.

### 3.6. Stabilisation

Stabilisation of the athlete, which refers to the athlete’s starting position and the control of compensations during maximal passive movements, is a fundamental phase of the ROM-SPORT I battery procedure in order to obtain an accurate measurement. In this sense, and in relation to the pelvis, it has been shown that significant compensatory movements of the pelvis occur during hip and knee movements (sagittal (posterior and anterior tilting), frontal (hike/upward tilt or drop/downward tilting) and transversal planes (backwards or forwards)) [25,67,73,74,75].

Consequently, these compensatory movements that increase ROM could alter the validity (false negative) of the measurement obtained [57,67]. Moreover, the pelvic tilt starts right at the beginning of the movement and gradually increases. The task of the assistant evaluator is to ensure the correct stability of the initial position, of a specific body segment, or of the pelvis throughout the assessment manoeuvre in order to avoid or minimise any compensatory movements that could increase and bias the final value.

Therefore, knowledge of the athlete’s starting position and control of the compensations, especially by the assistant evaluator, is essential.

### 3.7. Criteria for the End of the Range of Motion

Following the scientific literature, the endpoint for each ROM test is determined by one or more of the following criteria: (1) The main evaluator is unable to continue the stretching manoeuvre due to increased resistance of the muscle being tested; (2) The athlete feels a strong but tolerable stretch, just before the onset of pain; (3) One or both evaluators (main and assistant) have detected a palpable and perceptible compensatory movement that could increase the ROM [57,68].

### 3.8. Measurement

At the end of the maximum passive movement, the main evaluator places the telescopic arm of the inclinometer on the longitudinal axis of the moving body segment (imaginary line of the lateral bisector (sagittal movements) or the anterior bisector (frontal movements) of the mobilised body segment). The main evaluator then measures the angle formed by the longitudinal axis of the mobilised body segment and the line of gravity at 0°. At least two measurements per ROM test (dominant and non-dominant) and body limb in randomised order are recommended to obtain a reliable measurement. The mean value for each test is considered the final (true) ROM value [57,68,76]. In cases where a deviation of more than 5% was observed in the ROM values between the two attempts of a ROM test, an additional attempt is made. The two most closely related trials are used to calculate the true ROM value, provided the deviation in the new trial is <5%. If this is not the case, the evaluator would have to check the procedure for possible errors or circumstances that could explain the deviation. There was a 10 s break between the two trials and 30 s break between the tests. The athletes were tested in sportswear and without shoes.

### 3.9. Notes

Based on the scientific literature and the authors’ extensive previous experience, certain assessment skills have been reinforced in detail at the different phases of the procedure of each test, which influence the effectiveness of the procedure and the measurement result.

In summary, compared to other ROM assessment procedures, the use of the highlights of the ROM-SPORT I battery procedure contributes to a very fast implementation of the ROM test. The test duration of the ROM-SPORT I battery varies between about 15 (shortened version) and 20 (extended version) minutes. If the warm-up phase is omitted (e.g., in some clinical contexts), the duration is reduced to 7–11 min, although variability between sessions may increase by ±2°–4°. The estimated time for testing ROM with a telescopic arm inclinometer is 1 min per ROM test.

## 4. The Validity of the ROM-SPORT I Battery

With this in mind, all tests selected for the ROM-SPORT I battery have been validated by American medical organizations [58,77] and accepted by sports medicine and science handbooks [58,78] based on anatomical knowledge and extensive clinical experience (content validity or expert judge). The validity of an assessment test can be defined as the accuracy with which its measurement matches the true value, i.e., the extent to which it fulfils its objective [55]. Previous studies have demonstrated criterion validity of the gold standard [67,79,80,81]. In particular, control of compensatory movements or end-of-motion criteria are key elements of good validity.

After the evaluation of ROM, however, there is another important aspect to consider that is closely related to the concept of validity: The interpretation of the results is obtained on the basis of the values of a norm (normative validity) and a criterion (criterion validity). Normative validity values are used to compare the measurement of a ROM test with the normal values of a specific population or the flexibility profile of a sport. It is important to establish values that allow sports professionals to determine an athlete’s performance potential. In this sense, the research group “E0B5-07 Musculoskeletal System and Sport” of the University of Murcia has determined the flexibility profile using the ROM-SPORT I battery in soccer [49,82,83], futsal [37,44,84,85], basketball [86], handball [37,87], inline hockey [88,89], duathlon [90], taekwondo [91,92], and equestrian athletes [93].

Criterion-related values (criterion validity) refer to a cut-off value that indicates a high risk of a specific type of injury. It is important to establish values that allow clinicians, physiotherapists, and sports physicians to identify athletes at risk of injury. This knowledge would enable the early application of individualised training programmes to improve (athletes with borderline values or at high risk of injury) or maintain (athletes with normal values or at low risk of injury) baseline ROM levels and thus minimise the impact of one of the most important risk factors on the likelihood of lower limb injury. Table 1 shows the evidence-based cut-off values of several of the 11 tests selected for ROM-SPORT I battery that can be used to classify the ROM measures as restricted/high risk of injury or normal/low risk of injury. It should be noted, however, that there is no consensus on what constitutes normal and “high injury risk” values for some of the ROM measures. For this reason, several values for each category and measurement are given in Table 1, depending on the study, and should therefore be interpreted with some caution.

## 5. Reliability of the ROM-SPORT I Battery

Reliability is about the reproducibility of a measurement, i.e., whether the application of the assessment method can consistently produce the same results under the same conditions. In clinical or sports assessments, the reliability of a measurement is determined by human factors (the evaluator’s experience or training in administering the test, variations in assessment methodology, and individual-related variability) and/or the instrument used. Based on the fact that the most commonly used instruments for estimating ROM (goniometer and inclinometer) have proven to be reliable [57,69,76], the reliability of lower limb ROM measurements depends mainly on human factors [76]. Two different aspects of human reliability-related factors should be discussed before considering an appropriate measure for sports and clinical purposes: inter-evaluator reliability and intra- evaluator reliability [102]. Intra-evaluator reliability provides information on the extent to which multiple measurements taken at different times for the same test by the same evaluator are similar [103]. Intra-evaluator reliability can be determined if there are short (generally less than 3 h: intra-session) or long (generally more than 24 h: inter-session) intervals between test sessions. Longer periods between assessments (e.g., two weeks) are very important in clinical and sports contexts as they allow clinicians and sports professionals to monitor the performance or health status of their athletes and make informed decisions about whether a real change has occurred between testing sessions following the application of a training intervention [55]. The studies that analysed the intra-evaluator reliability of the values from the ROM-SPORT I battery using long time intervals between sessions (>24 h) showed moderate to high inter-session reliability scores (Table 2). Therefore, researchers, clinicians, and sports professionals can be 95% confident that a change between two measurements of more than 4°–7° for the ROM values from the ROM-SPORT I battery is likely to indicate a real change (determined by the statistical minimal detectable change with a 95% confidence interval [MDC_95%_]).

The key element for the reliability of the measurement obtained with the ROM-SPORT I battery is the use of the inclinometer as a measuring tool (Table 2). This fact, confirmed in several studies using other methods, has shown that the inclinometer is extremely reliable for lower limb ROM measurements (intraclass correlation index (ICC) > 0.90) and does not have the disadvantage of the goniometer, which requires precise positioning of its arms while the goniometer is moved simultaneously with the limb [58,106]. Finally, in contrast to other, more sophisticated devices (video cameras or isokinetic dynamometers), the cost of an inclinometer is relatively low (about 150 €).

For an accurate assessment of ROM, athletes should not have performed any vigorous physical activity in the previous 48 h. In addition, practical familiarization with the tests and a warm-up before athletes perform the tests will improve the accuracy of the measurements. This also applies to training examiners with the tests and the ROM-SPORT I battery procedure.

## 6. Practical Applications of the ROM-SPORT I Battery

The ROM-SPORT I battery can also be used in sports, clinical, and research settings with the following objectives:

− To accurately quantify the ROM measurements of the hip, knee and ankle, and increased tolerance to stretch [107,108].
− To determine the possibility of physical–technical sport performance in athletes who participate in sports that require technical skills with a high ROM (e.g., taekwondo, diving, figure skating, and gymnastics) [36,38,109].
− To identify athletes with muscle tightness that results in limited ROM (or high risk of injury), especially in soft tissue injuries [44,88].
− To quantify the effectiveness of intervention programs or significant chronic ROM changes (e.g., stretching and foam rolling intervention) aimed at maintaining or improving muscle flexibility in both healthy and injured individuals [110].
− In physical therapy processes, to determine whether the ROM of the injured joint has been fully restored, which may contribute to a safe return to play (athletes) or activities of daily living (general population) [111].

For future research, it would be necessary for more studies to use the ROM-SPORT I battery to define the ROM cut-off values for different groups in terms of sex, age, physical condition, type of sport, etc., to identify what values would be considered normal and which ones are considered of high risk for injury.

## 7. Conclusions

The eleven tests of the ROM-SPORT I battery described here seem to be the most appropriate for assessing the ROM of the major joints of the lower limbs. The use of the telescopic arm of the inclinometer and the Lumbosant, as well as the competence of the evaluators, are key elements for accurate, reproducible, and rapid measurement. The ROM-SPORT I battery has criterion-related values to identify individuals at high risk for a specific type of injury.

## Figures and Tables

**Table 1:** High risk of injury cut-off values of the tests selected for the ROM-SPORT I battery.

ROM Test	Restricted ROM/High Risk of Injury (Predictive Validity)
Hip flexion with the knee extended	≤68° [19] ≤70–71° [14,25,94] ≤88° [18]
Hip flexion with the knee flexed	≤111° [95] ≤114° [96] ≤120° [59]
Hip extension with the knee relaxed	<0° [97,98] ≤13° [19]
Hip adduction with the 90° hip flexion	≤19° [95] ≤20° [98] ≤26° [93]
Hip abduction with the knee extended	≤28° [19] ≤45° [10,58,77]
Abduction with the 90° hip flexion	≤50° [58] ≤80° [77]
Hip internal rotation	≤23° [23] ≤28–30° [22,24,29,99]
Hip external rotation	≤24–26° [9,13,95]
Knee flexion	≤120–121° [18,100] ≤128° [93] ≤132° [19,96]
Ankle dorsi-flexion with the knee extended	≤17° [19]
Ankle dorsi-flexion with the knee flexed	≤28° [19] ≤35–37° [15,16] ≤45° [101]

°: degrees; numbers in brackets represent the specific reference that has reported the cut-off value.

**Table 2:** Investigative studies that examined the intra-evaluator reliability of the assessment tests selected for ROM-SPORT I battery over a longer period of time (>one day).

Reference/Sample	Test	Human and Material Resources	Procedure	N° of Measurements and Time Interval	Results
Ayala et al. (2012) [104] M (*n* = 70) Recreational athletes	Hip flexion with the knee extended	2 evaluators Inclinometer Lumbosant	5 min cicloergometer and stretching 2 trials, mean	3 sessions 4 weeks	SEM = 4.1° ICC = 0.88
Cejudo et al. (2014) [66] M (*n* = 24) F (*n* = 26) Recreational athletes	Ankle dorsiflexion with the knee flexed	One evaluator Inclinometer	No warm-up 2 trials, mean	3 sessions 2 weeks	SEM = 1.3° MDC_95%_ = 3.8° ICC = 0.95
Cejudo et al. (2015) [68] M (*n* = 60) F (*n* = 30) Futsal players Handball players	(a) Hip flexion with the knee flexed (b) Hip flexion with the knee extended (c) Hip extension with the knee relaxed (d) Hip abduction with the knee extended (e) Knee flexion	2 evaluators Inclinometer Lumbosant	5 min jogging and stretching 2 trials, mean	3 sessions 2 weeks	(a) SEM = 2.5°; MDC_95%_ = 6.9° (b) SEM = 1.9°; MDC_95%_ = 5.3° (c) SEM = 1.3°; MDC_95%_ = 3.6° (d) SEM = 1.8°; MDC_95%_ = 5.0° (e) SEM = 2.8°; MDC_95%_ = 7.8°
Cejudo et al. (2015) [105] M (*n* = 25) F (*n* = 25) Recreational athletes	(a) Hip abduction with the knee extended (b) Hip abduction with the 90° hip flexion	Two evaluators Inclinometer	No warm-up 2 trials, mean	4 sessions 2 weeks	(a) SEM = 2.0°; MDC_95%_ = 5.5° (b) SEM = 2.1°; MDC95 = 5.8°
Cejudo et al. (unpublished data) M (*n* = 40) F (*n* = 18) Recreational athletes	(a) Hip adduction with the 90° hip flexion (b) Hip internal rotation with the knee flexed (c) Hip external rotation with the knee flexed	Two evaluators Inclinometer	No warm-up 2 trials, mean	4 sessions 6–8 day apart	(a) SEM = 1.8°; MDC_95%_ = 4.5° (b) SEM = 1.9°; MDC_95%_ = 5.5° (c) SEM = 2.1°; MDC_95%_ = 5.9°

SEM: standard error of the measure; MDC_95%_: minimal detectable change at 95% confidence interval; ICC: intraclass correlation index; SE: standard error; M: male; F: female.
